# Feasibility of Laser Communication Beacon Light Compressed Sensing

**DOI:** 10.3390/s20247257

**Published:** 2020-12-18

**Authors:** Zhen Wang, Shijie Gao, Lei Sheng

**Affiliations:** 1 Changchun Institute of Optics, Fine Mechanics and Physics, Chinese Academy of Sciences, Changchun 130033, China; wangzhen164@mails.ucas.edu.cn (Z.W.); shenglei@ciomp.ac.cn (L.S.); 2 University of Chinese Academy of Sciences, Beijing 100049, China

**Keywords:** compressed sensing, laser communication, deep learning, measurement matrixes, beacon light tracking, light-spot images storage

## Abstract

The Compressed Sensing (CS) camera can compress images in real time without consuming computing resources. Applying CS theory in the Laser Communication (LC) system can minimize the assumed transmission bandwidth (normally from a satellite to a ground station) and minimize the storage costs of beacon light-spot images; this can save more than ten times the typical bandwidth or storage space. However, the CS compressive process affects the light-spot tracking and key parameters in the images. In this study, we quantitatively explored the feasibility of the CS technique to capture light-spots in LC systems. We redesigned the measurement matrix to adapt to the requirement of light-tracking. We established a succinct structured deep network, the Compressed Sensing Denoising Center Net (CSD-Center Net) for denoising tracking computation from compressed image information. A series of simulations was made to test the performance of information preservation in beacon light spot image storage. With the consideration of CS ratio and application scenarios, coupled with CSD-Center Net and standard centroid, CS can achieve the tracking function well. The information preserved in compressed information correlates with the CS ratio; higher CS ratio can preserve more details. In fact, when the data rate is up than 10%, the accuracy could meet the requirements what we need in most application scenarios.

## 1. Introduction

The use of laser beams to carry information through free space is a popular technique due to its directivity, power dissipation, high bandwidth and high data rate [1,2,3,4,5]. The effects of atmospheric turbulence are highly complex [6,7,8,9]; optical signals and real-time beacon light-spot images reflect such effects in communication applications. Light spot images transmit and storage are challenging because of the high resolution and high frame frequency of the image sensor, which generates several gigabytes of data per second. It is difficult to accomplish real-time compression over such a magnitude of data while preserving computing resources. Likewise, it is extremely challenging to transmit (especially from satellite to ground station) and store light-spot images due to the high data volume. To the best of our knowledge, however, there has been no previous research focused specifically on these issues. Typically, in Laser Communication (LC) experiments, all the spot image information in the satellite is discarded and vast quantities of storage space are occupied to save the real-time images.

Compressed Sensing (CS) is a technique to acquire compressed images in real-time without consuming computing resources. This approach totally differs from traditional image compression. CS receives a compressed image directly from a special optical system and reconstructs the compressed information as required. Mathematically, the compressive projecting process can be abstracted as follows [10,11,12,13]:

y = Ax(1)

where ${x\in R}^{n}$ is the simple image in the traditional image sensor, ${y\in R}^{m}$ is the compressed data received from the CS camera and ${A\in R}^{n}$ is the “measurement matrix” or “project matrix”; Matrix A must satisfy the restricted isometric property (RIP), which guarantees the fewest possible measurements but with high probability of recovering the original signal [14]. CS reconstruction is a problem of reconstructing the unknown vector x after observing the m < n liner measurement, y, of its entry. We assume that the signal x here is k-sparse in some known basis, which is common for a photograph and that Ψ is the matrix transform x to this domain. Thus, x = Ψα and α is the transformed k-sparse signal. Next, x can be calculated as follows:

(2) $$\arg\;\min||\phi x|{|}_{0}\;s.t.\;y=\mathrm{Ax}=A\Psi \alpha =\Theta \alpha$$

where $|\left|\cdot \right|{|}_{0}$ denotes the $l_{0}$ norm and α=ϕx. Unfortunately, the $l_{0}$ norm problem is an NP-hard problem. There is an extended version:

(3) $$\arg\;\min||\phi x|{|}_{p}\;s.t.\;y=\mathrm{Ax}=\Theta \alpha$$

where $|\left|\cdot \right|{|}_{p}$ denotes the $l_{0}$ norm. As per the Lagrange method:

(4) $$\arg\;\min\;||\phi x|{|}_{p}+\lambda y-{\Theta \alpha ||}_{2}$$

Practically, it is a mature technology for this problem by use of traditional optimization or deep-learning methods [15,16,17,18,19,20,21,22,23,24,25,26].

Many CS imaging modules have been proposed in recent years. These modules project Equation (1) to optics systems. In one such study, a digital mirror array device was used to randomly project the image on a single sensor [27]. Successive random exposures were taken by randomly changing the digital mirror array. Other researchers [28] placed a random phase mask on a lens to randomly project the object on the array of sensors with fewer pixels. This captured compressed images in a single shot. Stern [29] proposed a linear sensor to capture compressed images and allow for faster acquisition of each frame compared to traditional scanning imaging systems. Ye et al. [30] proposed a code pattern design for multi-shot measurement. Other researchers built an optical model for the modeling and design of specifically tailored phase masks ensuring satisfactory contract-to-noise ratios [31]. An image reproduction scheme with an ultra-high-speed temporally compressive multi-aperture CMOS image sensor was demonstrated in another study [32]. In this system, because signals are modulated pixel-by-pixel during the capturing process, the maximum frame rate is defined only by the charge transfer speed and can thus exceed that of conventional ultra-high-speed cameras. Esteban et al. [33] proposed the use of aberrations to achieve effective single-shot compressive imaging. A CMOS approach to obtain compressed-domain image was established in another study [34], where the compressive module is achieved by an intelligent readout integrated circuit. Indeed, much more previous researchers have established compressed imaging systems; taking place of image sensors is practicable with workable support from these modules. 

CS techniques are popular in some special areas, for example, remote sense [35,36,37], radar [37,38,39] and Magnetic Resonance Imaging (MRI) [40,41,42]. These scenarios always preoccupied with the contradiction of huge volume of raw data and data compression computing consumption or the unacceptable sensing time. The compressing process of CS is succinct and low-spending in power and time, which conforms to the demand of scenarios described above. Actually, beacon light image belongs to remote sense but we need to acquire the centroid for spot tracking in real-time; the problem became even more complicated.

**Figure 1:**
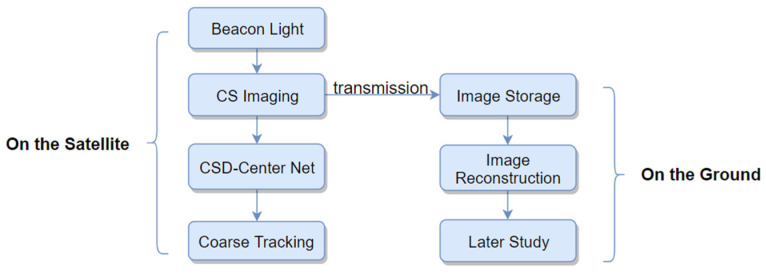
Laser Communication (LC) tracking system utilizing Compressed Sensing (CS): two algorithms, CSD-Center, image reconstruction deployed for various functions.

A system utilizing CS imaging to take place of image sensors is illustrated in Figure 1. The CS performs two necessary functions: light coarse tracking and light-spot image storage. This requires obtaining the plot center directly from compressed information and maintaining sufficient atmospheric details for subsequent analysis. However, obtaining the plot center from the compressed information is challenging because the image reconstruction requires burdensome calculations. We approached this problem by redesigning the measurement matrix and building a succinct structured deep network, the CSD-Center Net, which can compute light-plot centers directly and swiftly while requiring relatively little computation, as discussed in greater detail below. As for image storage, compressed images restored in real-time must preserve sufficient details and crucial information, for example, the refractive index structure constant ($C_{n}^{2}$) and angle-of-arrival fluctuation [9,43]. It is critical to quantify the effects that compression processes with different ratios exert upon various parameters. The next two sections will discuss the beacon light tracking and compression performance respectively.

## 2. Beacon Light Tracking and CSD-Center Net

For an ordinary image, it is easy to access the centroid $\left(C_{h},{\;C}_{v}\right)$,

(5) $$\begin{array}{cc} C_{h} & =\;\frac{\sum_{i,j=0}^{n}x_{i,j}w_{i}}{\sum_{i,j=0}^{n}x_{i,j}}\\ & =\mathrm{Sum}\left({\mathrm{Xw}}^{T}\right)/\mathrm{Sum}\left(X\right)\\ & =\mathrm{Sum}\left[{\left(x_{1},x_{2},\ldots ,x_{n}\right)}^{T}w^{T}\right]/\mathrm{Sum}{\left(x_{1},x_{2},\ldots ,{\;x}_{n}\right)}^{T}\\ & =\mathrm{Sum}\left(x_{1}w^{T},x_{2}w^{T},\ldots ,x_{n}w^{T}\right)/\mathrm{Sum}\left[\mathrm{Sum}\left(x_{1}\right),\mathrm{Sum}\left(x_{2}\right),\ldots ,\mathrm{Sum}\left(x_{n}\right)\right] \end{array}$$

(6) $$\begin{array}{cc} C_{v} & =\frac{\sum_{i,j=0}^{n}w_{i}x_{i,j}}{\sum_{i,j=0}^{n}x_{i,j}}\\ & =\mathrm{Sum}\left(\mathrm{wX}\right)/\mathrm{Sum}\left(X\right)\\ & =\mathrm{Sum}\left[w\left(x_{1},x_{2},\ldots ,x_{n}\right)\right]/\mathrm{Sum}\left(x_{1},x_{2},\ldots ,x_{n}\right)\\ & =\mathrm{Sum}\left({\mathrm{wx}}_{1},{\mathrm{wx}}_{2},\ldots ,{\mathrm{wx}}_{n}\right)/\mathrm{Sum}\left[\mathrm{Sum}\left(x_{1}\right),\mathrm{Sum}\left(x_{2}\right),\ldots ,\mathrm{Sum}\left(x_{n}\right)\right], \end{array}$$

where w is the row vector of position weight, $x_{k}$ denotes the k-th row in $C_{h}$ (k-th column in $C_{v}$) of X and Sum(·) sums all elements of the vector or matrix. To get $C_{v}$, we have to get $\mathrm{Sum}\left(x_{k},w\right)$ and Sum($x_{k}$) through measured matrix y respectively; matrix A in Equation (1) has a great influence on this issue and proper A can promote solution of the problem. As we mentioned before, traditional measurement matrix A must satisfy RIP:

(7) $$\left(1-\sigma \right)\left|\left|c\right|{|}_{2}\leq \right|\left|\mathrm{Ac}\right|{|}_{2}\;\left(1\;+\;\sigma \right)|\left|c\right|{|}_{2}$$

where $|\left|\cdot \right|{|}_{2}$ is $l_{2}$ norm, c is a sparse vector and σ ∈ (0, 1). Gaussian random matrix, Toeplitz measurement matrix, cyclic matrix, Bernoulli’s matrix and so forth are typical matrixes meet the requirement. However, there are many inspiring chosen, for example, a Gaussian random matrix A (u, δ), we have:

y = Ax

which can be write as:

$$\begin{array}{cc} y_{1}\; & ={\;a}_{11}x_{11}\;+{\;a}_{12}x_{12}\;+\;\ldots \;+{\;a}_{1n}x_{1n}\\ y_{2}\; & ={\;a}_{21}x_{11}\;+{\;a}_{22}x_{12}\;+\;\ldots \;+{\;a}_{2n}x_{1n}\\ \cdots & \\ y_{m}\; & ={\;a}_{m1}x_{11}\;+{\;a}_{m2}x_{12}\;+\;\ldots \;+{\;a}_{\mathrm{mn}}x_{1n}. \end{array}$$

Actually, for $\mathrm{Sum}\left(x_{k}w\right)$ and $\mathrm{Sum}\left(x_{k}\right)$ in Equations (5) and (6), we have:

$$\begin{array}{cc} \mathrm{Sum}\left(x_{k}w\right)\; & ={\;w}_{1}x_{k1}\;+{\;w}_{2}x_{k2}\;+\;\ldots \;+{\;w}_{n}x_{\mathrm{kn}}\\ \mathrm{Sum}\left(x_{k}\right) & ={\;x}_{k1}\;+{\;x}_{k2}\;+x_{k3}+\;\ldots \;+{\;x}_{\mathrm{kn}} \end{array}$$

then Sum($y_{1},\;y_{2},\;\ldots ,\;y_{m}$) ≈ $m\;u\;\mathrm{Sum}\left(x_{k}\right)$ if the m is large enough, which requires high resolution ratio of image sensor and high CS ratio to preserve ${\mathrm{Sum}(a}_{k,i}\;|\;k=1,2,\ldots ,m;i<n)\approx \mathrm{mE}\left(A\right)=m\;u$. Unfortunately, we cannot acquire $\mathrm{Sum}\left(x_{k}w\right)$ directly with these measure matrix. If we add two extra row vector to measure matrix A, then we have:

$$\begin{array}{cc} y_{1}\;= & a_{11}x_{11}\;+{\;a}_{12}x_{12}\;+\;\ldots \;+{\;a}_{1n}x_{1n}\\ y_{2}\;= & a_{21}x_{11}\;+{\;a}_{22}x_{12}\;+\;\ldots \;+{\;a}_{2n}x_{1n}\\ \cdots & \\ y_{m}\;= & a_{m1}x_{11}\;+{\;a}_{m2}x_{12}\;+\;\ldots \;+{\;a}_{\mathrm{mn}}x_{1n}\\ y_{m+1}\;= & {\beta w}_{1}x_{11}\;+{\beta w}_{2}x_{12}\;+\;\ldots \;+{\beta w}_{n}x_{1n}\\ y_{m+2}\;= & {\alpha x}_{11}\;+{\alpha x}_{12}\;+\alpha x_{13}\;\ldots \;+{\alpha x}_{1n} \end{array}$$

then:

(8) $$\begin{array}{cc} C_{v}\; & =\frac{\frac{\alpha }{\beta }\mathrm{Sum}\left(y_{m+1,1},{\;y}_{m+1,2},\;\ldots ,{\;y}_{m+1,n}\right)}{\mathrm{Sum}\left(y_{m+2,1},{\;y}_{m+2,2},\;\ldots ,{\;y}_{m+2,n}\right)}\\ C_{h}\; & =\frac{\mathrm{Sum}\left(w_{1}y_{m+2,1},{\;w}_{2}y_{m+2,2},\;\ldots ,{\;w}_{n}y_{m+2,n}\right)}{\mathrm{Sum}\left(y_{m+2,1},{\;y}_{m+2,2},\;\ldots ,{\;y}_{m+2,n}\right).} \end{array}$$

However, when we are applying cyclic matrix as measurement matrix [44]:

(9) $$A=\;\left(\begin{array}{cccc} a_{n} & a_{n-1} & \cdots & a_{1}\\ a_{1} & a_{n} & \cdots & a_{n-1}\\ \vdots & \vdots & \ddots & \vdots \\ a_{n-1} & a_{n-2} & \cdots & a_{n} \end{array}\right)$$

then we have $\mathrm{Sum}\left(y\right)\;=\;\mathrm{mSum}\left(t\right)\mathrm{Sum}\left(x_{k}\right)$ and $y_{m+2}$ is not required.

To make full use of measurement matrix, we redesigned an m-rows’ measurement matrix using a deep learning network. In this network, the rows of measure matrix can be considered as m non-overlapping filters respectively and the sampling process can be considered as a convolutional layer. This means the extra rows using for centroid are utilized. Notice, the last two rows are fixed and there are m − 2 row need training. Which means:

$$\begin{array}{cc} y_{m-1}\; & =\;{\beta w}_{1}x_{11}\;+{\beta w}_{2}x_{12}\;+\;\ldots \;+{\beta w}_{n}x_{1n}\\ y_{m}\; & =\;{\alpha x}_{11}\;+{\alpha x}_{12}\;+\alpha x_{13}\;\ldots \;+{\alpha x}_{1n} \end{array}$$

combined with the RIP constraint of A, the loss function can be defined as:

(10) $$\mathrm{loss}=\;|\left|\left({\left||S_{\mathrm{out}}|\right|}_{2}-\;{\left||S_{\mathrm{in}}|\right|}_{2}\right)\right|{|}_{2}$$

where $S_{\mathrm{in}}{\in R}^{n}$, $S_{\mathrm{out}}{\in R}^{m}$ are the input and output of the network, $S_{\mathrm{out}}=Y_{m-2}\cup \left(y_{m-1},y_{m}\right)$. Network can be trained by sparse vector. Unless otherwise specified, the measurement matrixes below are using the trained matrixes by this network.

In fact, light-spot images in experiment always contain noise and the approach we mentioned above acquiring centroid without considering interference factors. A signal ${x\in R}^{n}$ with noise can be expressed as:

(11) x = s +ε.

s is the useful part and ε is the noise. The most common way to eliminate noise is to transfer x to a specific domain where we can separate s and ε; it is natural to consider the similarity of CS and denoising. Actually, tradition reconstruction method and denoising method always using same transform matrixes, for example, wavelet and DCT transform matrix and so forth. In such a domain, s always reflected to the strong signal on non-zero elements and ε always reflects to the small fluctuation on all elements. When we talked about CS, the fluctuation can approximately be dropped while removing the fluctuation is the means (or purpose) to the denoising. Practically, in some extent, the fluctuation limits the precision of CS reconstruction.

**Figure 2:**
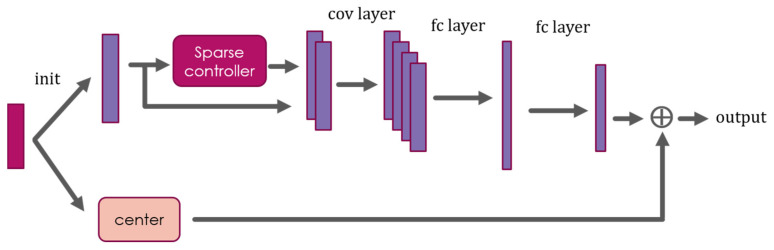
Proposed CSD-Center Net framework with CS process: convolutional layer and full connected layer were abbreviated with ‘cov layer’ and ‘fc layer,’ respectively.

We constructed a CNN network, dubbed CSD-Center Net, to calculate centroid with adjustable denoising element. The network diagram is presented in Figure 2. It is an improvement on the original Le-Net [45], from which we removed the pooling layer and reduced convolution layer to reduce the algorithm complexity and guarantee real-time performance. The bottom branch in Figure 2 is the standard centroid acquired from measurement matrix. In the upper branch, we are using a sparse controller to preserve the sparsity. This branch can be trained to acquire the influence of useless signals to the standard centroid. The sparse controller can be denoising model or sparsity model, only if it can preserve the real-time performance and signals sparsity. In this paper, we are using a fixed and well-trained ISTA-Net phase there. Actually, input of the network can be the whole image or row by row, which influenced the training process and the output, (C_sh_, C_sv_) or (Sum(w·x_i_), Sum(x_i_)), respectively. However, whole image pattern is more stable than row by row. The overall loss function is trivial:

(12) $$\mathrm{Loss}=\;{\left||C_{h}\;-{\;C}_{\mathrm{sh}}|\right|}_{2}\;+\;{\left||C_{v}\;-{\;C}_{\mathrm{sv}}|\right|}_{2}.$$

To train the CSD-Center Net and explore the performance of tracking and storage, we generate a targeted dataset, which contains distorted light-spot images with floating position, while the existing datasets open accessed are not available to this issue. The normal distorted images are generated by power spectrum inversion, which simulated the whole light propagation process and splits the process into two parts: one irrelevant to the refractive index in a vacuum and another relevant to the phase modulation. There are two approximations at work here: that the refractive index fluctuations are relatively small and that the two steps are independent. We applied a multi-layer phase screen to simulate the influence of the atmosphere on optical fields with phases generated under Kolmogorov theory and a von Karman power spectrum [46,47]. Fresnel diffraction theory was used to investigate the light propagation process in a vacuum, where the refractive index structure constant $C_{n}^{2}$ = 1 × 10^−14^ m^−2/3^ and the transmitting distance is 5 km. As described above, the compressed images received in the receiver were simulated by normal distorted images via Equation (1). The noise is adjustable according to the application environments.

To train this network, we divided 10,000 distorted images into 25 groups; different levels of noise was added to each group. 250 images, 10 images per group, are used to test the effects of CSD-Center Net. In fact, the training dataset can be more targeted than this sample according to the application scenarios. The speed of the network are checked on a laptop with Intel Core i5-3230 CPU. After training, the CSD-Center Net can complete the necessary computation of a 1 megapixels image in less than 1 millisecond—thus, it can track a light spot across more than one thousand frames per second. Before beginning, certain operational steps are necessary for the proper initialization.

Input Initialization: Compressed signals correlate to different sampling rates; each groups of signals must be trained individually based on the different sampling rates because of the dimension mismatch of input vectors y. To normalize the network input, we established an initialization for the different rates with a liner mapping matrix, denoted by $A_{\mathrm{init}}$, which can be computed by solving a least squares problem: $A_{\mathrm{init}}\;=\;\arg\;\min\;||\mathrm{AY}-X|{|}_{2}^{2}\;={\;\mathrm{XY}}^{T}{\left({\mathrm{YY}}^{T}\right)}^{-1}$. The liner mapping process is $S_{\mathrm{in}}=A_{\mathrm{init}}Y$ and input dimension of vectors was mapped from $R^{m}$ to $R^{n}$ Any input CS measurement is suitable for the network [22].

**Figure 3:**
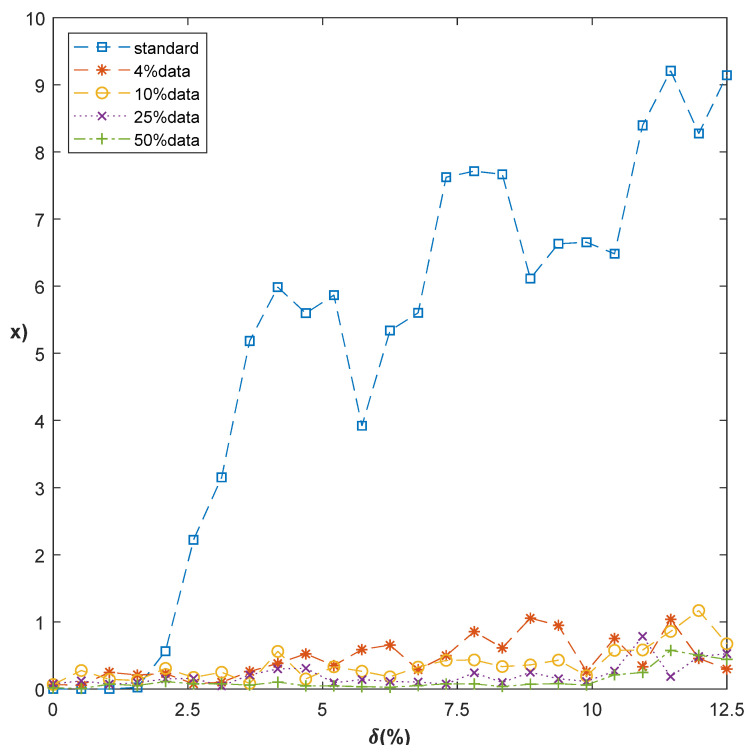
Tracking error of standard centroid and CSD-Center Net in noisy condition.

Figure 3 is the mean effects under different CS ratios of the network. As we can see, standard centroid error Δ acquired through Equation (8) effects extremely good in comparatively idea situations, that is, δ ≤2. With the increasing of the δ, standard centroid suffers a rapid deterioration. In this case, CSD-Center Net effectively lower the influence of noise, which makes the error less than 1 pixel even in higher levels of noise. As expected, the CS ratio affects the centroid error; higher CS ratio is often more effective than lower one. In a LC system, if the tracking accuracy is e ≤ 2 urad and each pixel corresponds to 1urad, we can acquire the overall tracking accuracy. When δ ≤2, standard centroid performs almost perfectly and CSD-Center Net has the opposite effect. When  δ ≥2, we need to use the CSD-Center Net because of the deterioration of standard centroid. Choose the ratio according to requirement is important in this situation. We evaluate the performance of CSD-Center Net with the Δ≤0.5 pix, that is, e ≤ 0.25 urad, which encompasses 12.5% of the overall system precision. As we can see, the Δ of different data rate started to be bad (e > 0.25) when the value of δ is over 7.5, 10.75 and 10.75 to 4%, 10% and 25% respectively. As for the 50% data rate, it is robust in the supplied scale. Practically, the deterioration speed of CSD-Center Net is relatively slow. Significantly, the precision of tracking using CSD-Center Net is not severely impacted by the measurement data rate. We can obtain the relatively good results using an extremely low data rate, for example, 4% data. This is a good feature for LC light tracking and real-time images storage; it means we can achieve the tracking function with fewer information. As a practical matter, coordinating the standard centroid and CSD-Center Net allow the light-tracking more efficiently.

## 3. Image Storage

CS imaging can track the spot in real-time with CSD-Center Net; it is necessary for a LC system. However, saving the details of light-spot image is actually the object of applying CS. In a LC system, it is normal that there always be a complex atmospheric processes in remotely distance between two ends of the communication. After the transmitting, the communication laser was distorted by the atmospheric turbulence. Light-spot images display the light intensity distribution of the communication laser. Practically, to a certain extent, the real-time images can reflect the influence of atmosphere turbulence. To test the effectiveness of the image saving, we determined the parameters in a reconstructed image from four different algorithms for comparison: Irls [17], ISTA-Net [22] and Ols [20,21] and FCSR [23]. Images without noise but still with distortion of atmosphere turbulence are used for this controlled experiment. The parameter reconstruction performance with the four algorithms was summarized in Table 1. It is worth mentioning that the effects can be improved with the development of CS reconstruction algorithm.

**Figure 4:**
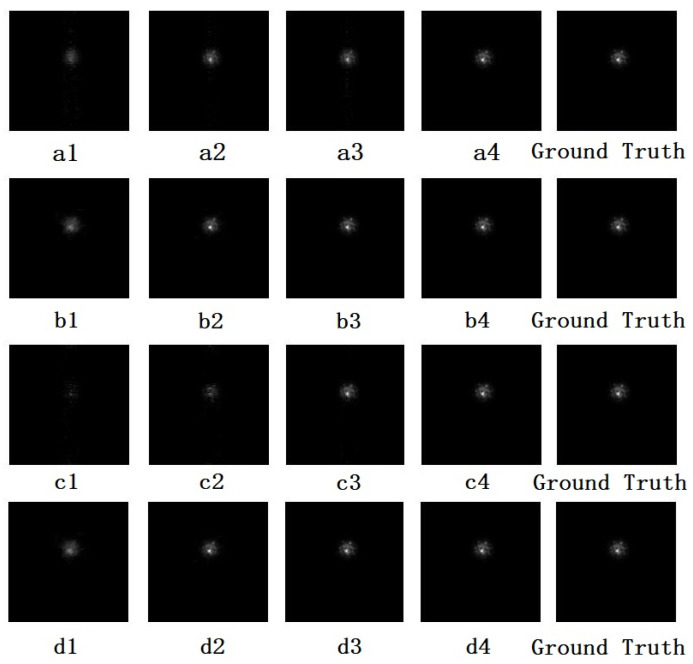
Reconstructed images of different rates and algorithms: (**a**, **b**, **c**, **d**) Irls, ISTA-Net, Ols and FCSR algorithm; (**1**, **2**, **3**, **4**) 4%, 10%, 25% and 50% CS rates, respectively.

The reconstructed image directly represents the reconstruction results. Figure 4 shows reconstructed images by different algorithms with different rates. The ISTA-Net and FCSR reconstruction algorithm performs well and shows no significant differences between the ground truth and the reconstructed image at a 10% CS rate with the naked eye. We also determined the average peak signal-to-noise ratio (PSNR) to quantitatively report reconstruction deviations over the test image (Table 1). Even at a 4% CS rate, the ISTA-Net algorithm has a PSNR of 47.5 dB. While producing a reconstructed image that is indistinguishable from the original by the naked eye, the algorithm saves over ten times the storage space of other algorithms in operating the CS. The reconstruction effects observed here indicate that the CS technique meets the requirements of LC systems. 

The refractive index structure constant ($C_{n}^{2}$) is one of the main parameters in atmospheric optics [43,48,49]. $C_{n}^{2}$ described the random variation of atmospheric structure and its physical parameters on various time and space scales. With the improvement of modern nonlinear dynamical [50,51], the atmosphere turbulence in various scale and various space-time plays an important role in saltation and its predictability of atmospheric processes. Therefore, it plays an important role in atmospheric turbulence and related problems. Fluctuations in the refractive index destroy the coherence of the light-wave leading to scintillation, laser beam drift and spread. In the LC system, long-term $C_{n}^{2}$ changes recorded in real time are significant in terms of communication quality. The $C_{n}^{2}$ losses shown in Table 1 are negligible compared to its fluctuations (normally more than one or two orders of magnitude in one day). Even at the extremely low compression rate of 4%, the loss is below 7%. At a CS rate of 10%, ISTA-Net has a loss below 1%.

**Figure 5:**
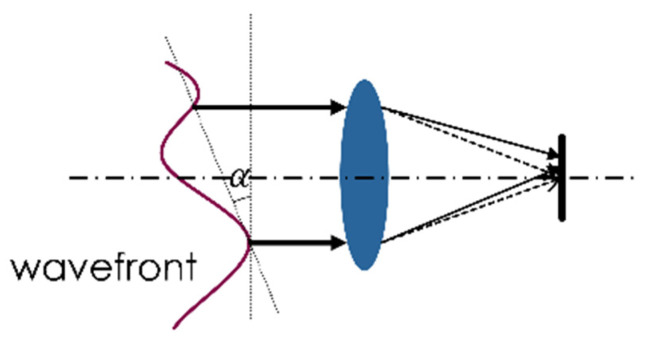
Center error formation of light beam in focal plane.

Random jitter in optical images occurs in the focal plane of the receiving terminal [43,52]. This so-called angle-of-arrival fluctuation affects communication effects and is the primary cause of tracking error. Practically, the angle-of-arrival fluctuation affects the LC tracking results significantly; a high frame frequency is generally used in the image sensor to track light plots with random fluctuation. Wavefront aberration, which is caused by atmosphere influence, decreases the wavefront inclination α and laser plot deviation of the sensor’s focal plane. Figure 5 shows the angle-of-arrival fluctuation formation. We can obtain α by measuring the variation of the difference Δx between the plot center and focal plane combined with pixel size p and focal length f:

(13) α= (Δx·p)/f

Although we can obtain the Δx approximately by using the redesigned measurement matrixes, we still used the standard way here in order to test the preservation performance of CS. We assumed that the CS system and traditional image sensor have same pixel size p and focal length f here. We can then measure the angle-of-arrival fluctuation by measuring the variation of the laser plot center from reconstructed images. 

**Figure 6:**
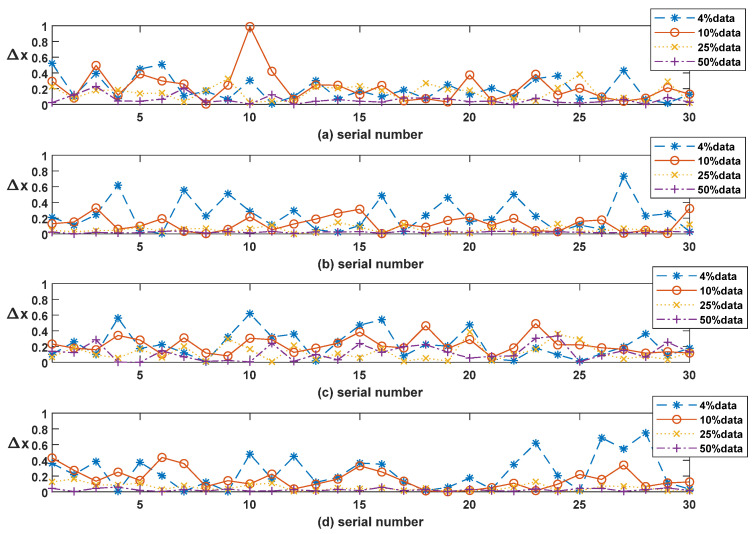
30-image center error line chart of different algorithms. (**a**) Irls; (**b**) ISTA-Net; (**c**) Ols; (**d**) FCSR.

Figure 6 shows a 30-image Δx line chart of different reconstruction algorithms. The overall system precision is represented by E(er) in Table 1. Apparently, The CS rates influence overall precision; higher CS rate can preserve more details. The average Δx deviations of 4%, 10%, 25% and 50% CS ratio are about 0.24, 0.13, 0.05 and 0.02 pixels respectively. From the statistics, extremely low CS rate, for example 4% data, result in inconvenient deviation. Actually, when the data rate is up to 10%, the loss always can be ignored in most application scenarios. This perception can be confirmed by PSNR, $C_{n}^{2}$ and random jitter (or Δx). Of course, some special decision such as the measurement matrixes, can help us decrease or remove the influence to some parameters.

## 4. Conclusions

In this study, we explored the feasibility of the CS technique for capturing light-spot in LC systems in order to minimize the storage and bandwidth cost of beacon light-spot images. In order to meet the requirement of light tracking, we redesigned the measurement matrixes. The redesigned matrixes can acquire the standard centroid directly. Standard centroid effects extremely well in comparatively idea situations. To achieve denoising tracking with compressed information, we built a succinct deep learning net, dubbed CSD-Center Net. The CSD-Center Net has low computational complexity, high precision and quick calculation speed. With the deterioration of image quality, CSD-Center Net effectively lower the influence of noise, which makes the error less than 1 pixel even in higher levels of noise. Standard centroid and CSD-Center Net are functionally complementary in different noisy environment. We can achieve high accuracy light-tracking by appropriate selection. 

We measured the effects CS to LC with special focus on quantitative parameters by simulating the entire light propagation and detection process. The CS was able to compress a beacon light-spot image in real-time without using computing resources and to reconstruct an image even at extremely low compressive rates (e.g., 4%) with PSNR values higher than 47 dB. The influence of CS to $C_{n}^{2}$ was found to be negligible compared to its own floating range. By contrast, the angle-of-arrival was found to be more sensitive to the CS rate if we do not use redesigned matrixes. A sufficiently small CS rate can help preserve more information in limited storage space when light-plot image information is necessary over extended periods of time; a larger CS rate can be set to preserve more precise or accurate light-spot information when necessary without consuming available bandwidth and storage resources. The interactions and mutual restriction among CS rate, data volume and parameter precision can be flexibly adjusted to suit different needs.

However, the analysis in this paper are based on a fairly idea condition in principle. There should be many practical difficulties in experiment such as optical manufacture, installation and adjustment, sampling noise and so forth. Parameters selection for different LC systems is critical; communication distance, optical antenna, coupled with pixels size all of this have great impact on CS rate. And that, in our opinion, how to reduce the impact of optical and electronics device and choose proper parameter and CS rate are the emphasis and difficulty in follow-up research. 

## Figures and Tables

**Table 1:** Average error of important parameters recovered from different algorithms.

Algorithm	CS Rate (%)	PSNR (dB)	$C_{n}^{2}\;(\%)$	Δx (pix)	E(er) (%)
Irls	4	41.3	16.76	0.1957	0.0979
	10	43.7	14.33	0.2161	0.1081
	25	50.4	5.87	0.1453	0.0727
	50	52.4	5.86	0.0588	0.0294
ISTA-Net	4	47.5	6.36	0.2372	0.1186
	10	50.6	0.96	0.1297	0.0649
	25	58.1	0.40	0.0535	0.0268
	50	60.8	0	0.0213	0.0107
Ols	4	41.1	12.74	0.2252	0.1126
	10	41.5	16.89	0.2188	0.1094
	25	42.9	19.34	0.1240	0.0620
	50	50.8	7.22	0.1220	0.0610
FCSR	4	46.3	8.41	0.2187	0.1094
	10	49.9	1.88	0.1337	0.0669
	25	55.7	0.57	0.0528	0.0264
	50	59.6	0.03	0.0364	0.0182
